# Supplementary material for: A genome-wide meta-analysis yields 46 new loci associating with biomarkers of iron homeostasis
Source: Commun Biol. 2021 Feb 3;4:156. doi: 10.1038/s42003-020-01575-z (PMC7859200; doi:10.1038/s42003-020-01575-z)
Supplement: Supplementary file 2 — Supplementary Information [file 42003_2020_1575_MOESM2_ESM.pdf]

Supplementary Figures for

A genome-wide association meta-analysis yields 46 novel loci associated with iron homeostasis biomarkers

Steven Bell [1,2] †, Andreas Stribolt Rigas [3] †, Magnus K. Magnusson [4,5] †*, Egil Ferkingstad [4] †, Elias Allara [1,2] †, Gyda Bjornsdottir [4], Anna Ramond [1,2,6], Erik Sørensen [3], Gisli Halldorsson [4], Dirk S. Paul [1,2], Kristoffer Sølvsten Burgdorf [3], Hannes P. Eggertsson [4], Joanna M.M. Howson [2], Lise Wegner Thørner [3], Snaedis Kristmundsdottir [4], William J Astle[1,2,7,8], Christian Erikstrup [9], Jon K. Sigurdsson [4], Dragana Vuckovic [1,8], Khoa Manh Dinh [9], Vinicius Tragante [4,10], Praveen Surendran [2,11], Ole Birger Vesterager Pedersen [12], Brynjar Vidarsson [13], Tao Jiang [1,2,8,] , Helene Paarup [14] , Pall T. Onundarson [5,15], Parsa Akbari [1,2,8], Kaspar René Nielsen [16], Sigrun H. Lund[4], Kristinn Juliusson[4], Magnus I. Magnusson[4], Michael L. Frigge[4], Asmundur Oddsson[4], Isleifur Olafsson [17], Stephen Kaptoge [1,2], Henrik Hjalgrim [18], Gudmundur Runarsson [13], Angela M. Wood [1,2], Ingileif Jonsdottir [4,5], Thomas Folkmann Hansen [19,20,21], Olof Sigurdardottir [22], Hreinn Stefansson [4], David Rye [23], DBDS Genomic Consortium, James E. Peters [2], David Westergaard [24], Hilma Holm [4], Nicole Soranzo [1,8,11], Karina Banasik [24], Gudmar Thorleifsson [4], Willem H Ouwehand [1,8,25,26], Unnur Thorsteinsdottir [4,5], David J Roberts [1,27,28], Patrick Sulem [4], Adam S Butterworth [1,2], Daniel Gudbjartsson [4,29], John Danesh [1,2,8], Søren Brunak [24], Emanuele Di Angelantonio [1,2,25] ‡*, Henrik Ullum [3] ‡*, Kari Stefansson [4,5] ‡*

† These authors contributed equally (co-first authors); ‡ These authors jointly supervised this work
***Corresponding authors:** Magnus K. Magnusson (magnus.magnusson@decode.is), Emanuele Di Angelantonio (ed303@medschl.cam.ac.uk), Henrik Ullum (Henrik.Ullum@regionh.dk), and Kari Stefansson (kstefans@decode.is).

For affiliations see cover page.

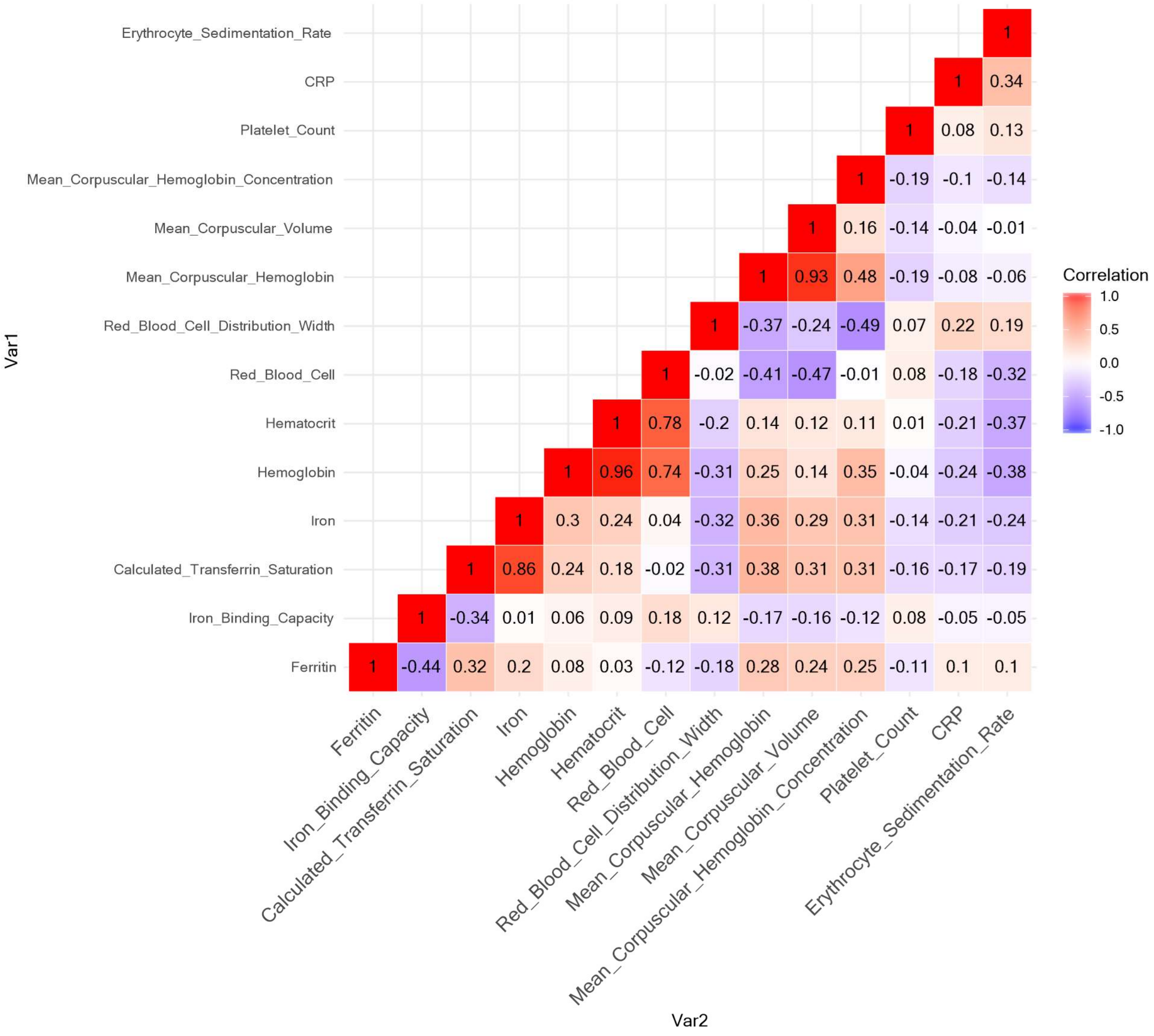

Supplementary Figure 1: Correlation between the iron biomarkers and selected other biomarkers (red blood cell indices, platelet count, erythrocyte sedimentation rate and C-reactive protein). The correlation between measured markers are shown and also indicated using a heatmap (color scheme shown to the right).

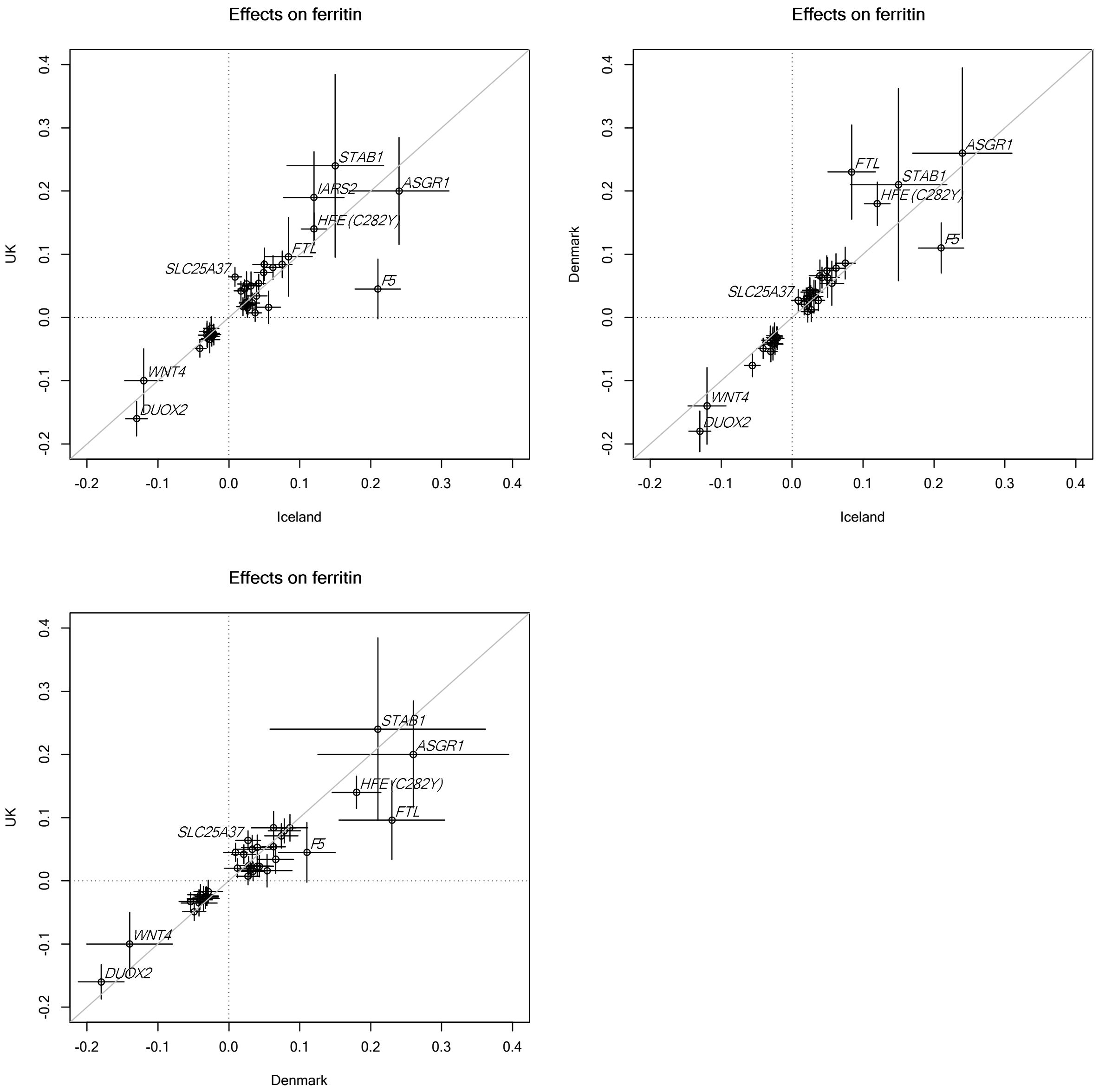

Supplementary Figure 2: Comparison of the effects on ferritin in Iceland, the UK and Denmark. Effects are shown in units of standard deviations. Crosses indicate 95% confidence intervals.

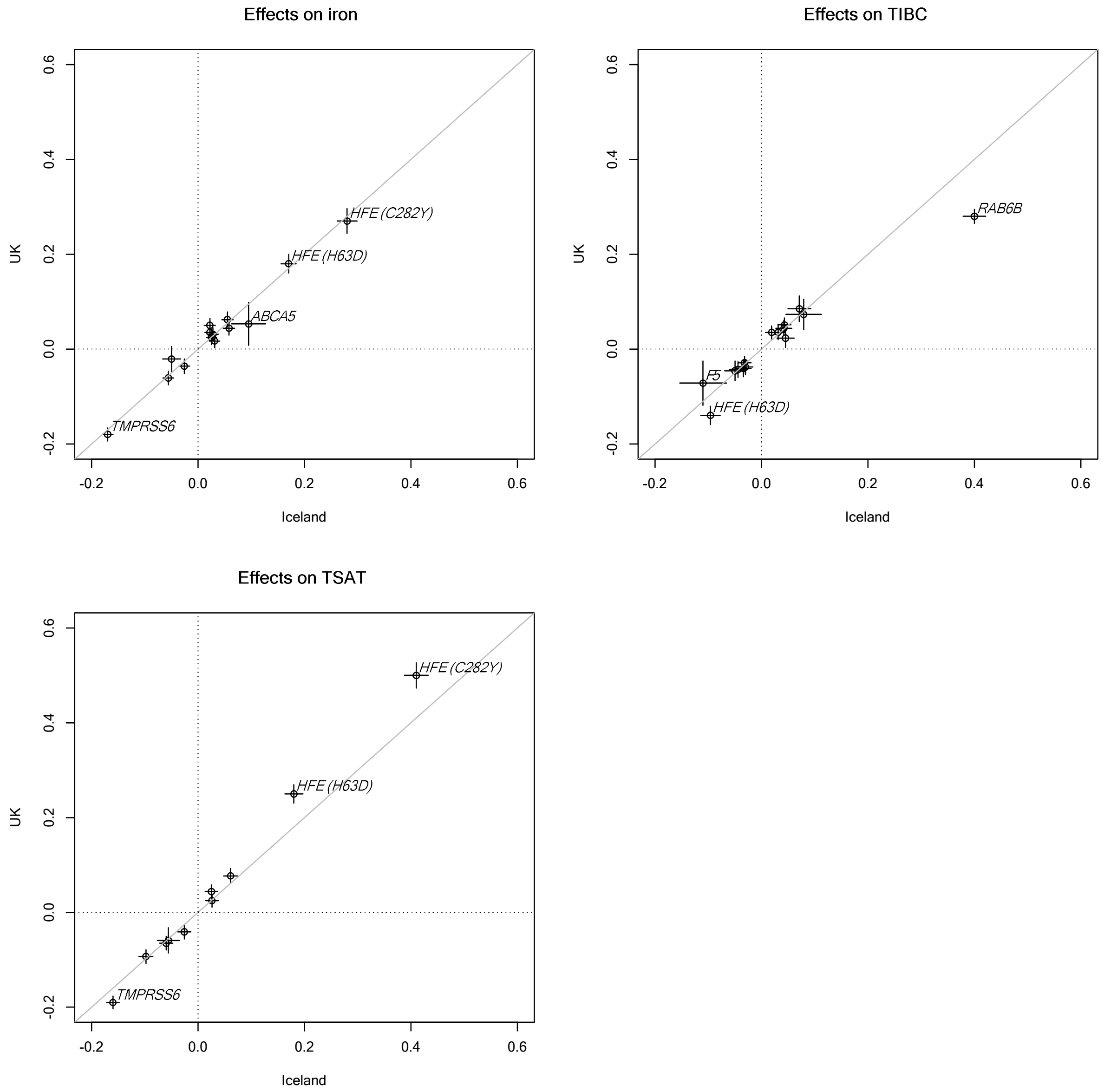

Supplementary Figure 3: Comparison of the effects on iron, TIBC and TSAT in Iceland and the UK. Effects are shown in units of standard deviations. Crosses indicate 95% confidence intervals.

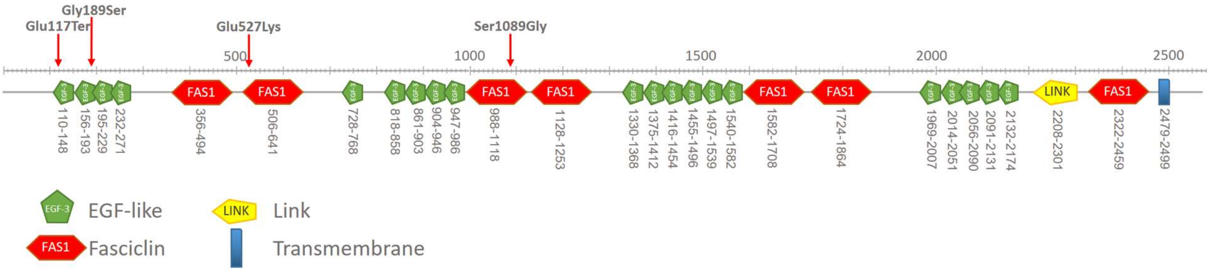

Supplementary Figure 4: ScanProsite results together with ProRule-based predicted intra-domain features of Stabilin-1 (STAB1). STAB1 encodes a large, transmembrane scavenger receptor. The protein contains seven fasciclin (FAS1/BIgH3) domains, 20 epidermal growth factor (EGF)-like domains, C-type lectin-like hyaluronan-binding Link (Link) domain and a transmembrane domain. The four coding variants associating with ferritin levels are represented by red arrows.

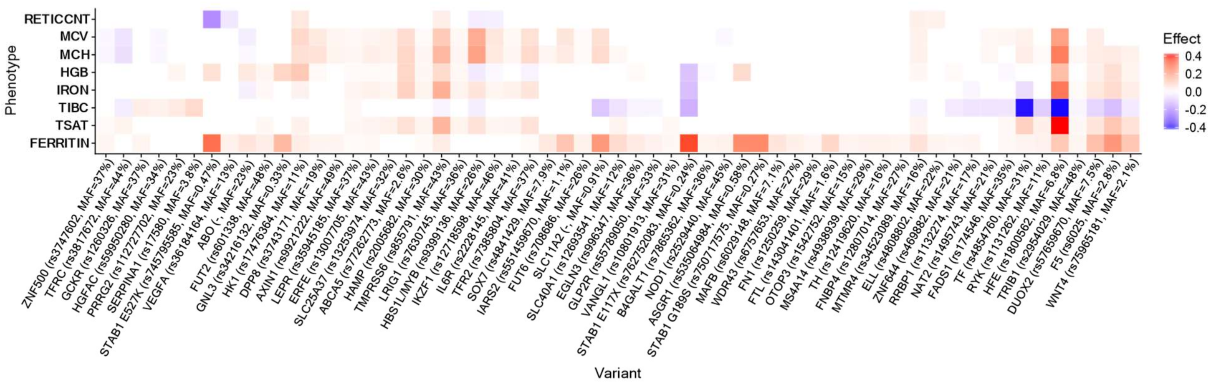

Supplementary Figure 5: Association of iron homeostasis variants with ferritin (N=172,764), total iron binding capacity (TIBC, N=95,314), transferrin saturation (TSAT, N=91,308), serum iron (N=123,314), hemoglobin (HGB, N=286,622), mean corpuscular hemoglobin (MCH, N=286,245), mean corpuscular volume (MCV, N=286,248) and reticulocyte count (RETICCNT, N=19,031) in the Icelandic data. Effects are in units of standard deviations and are shown for the allele having a positive effect on ferritin. Variants are ordered based on a hierarchical clustering algorithm. Effects that did not reach a false discovery rate (FDR) threshold of FDR<0.05 are shown as zero (white).

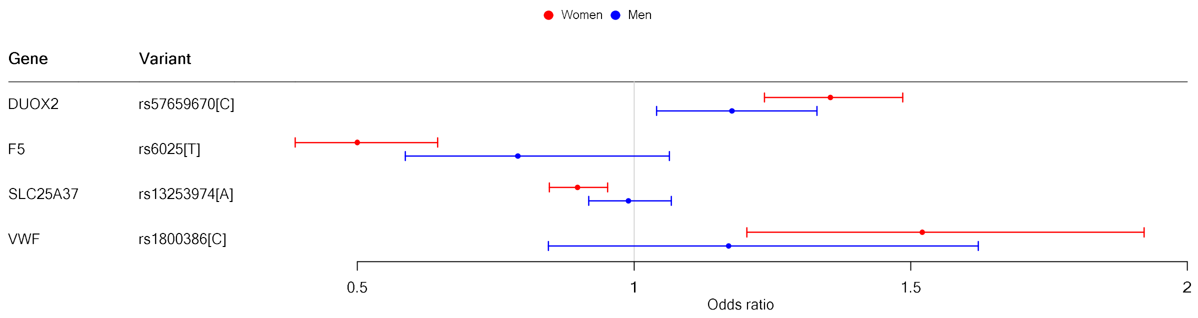

Supplementary Figure 6: Novel iron homeostasis variants showing sexual dimorphism for iron deficiency anemia (IDA). A forest plot showing the odds ratio for IDA (error bars showing 95% confidence intervals) for each of the genetic variants showing sexual dimorphism.

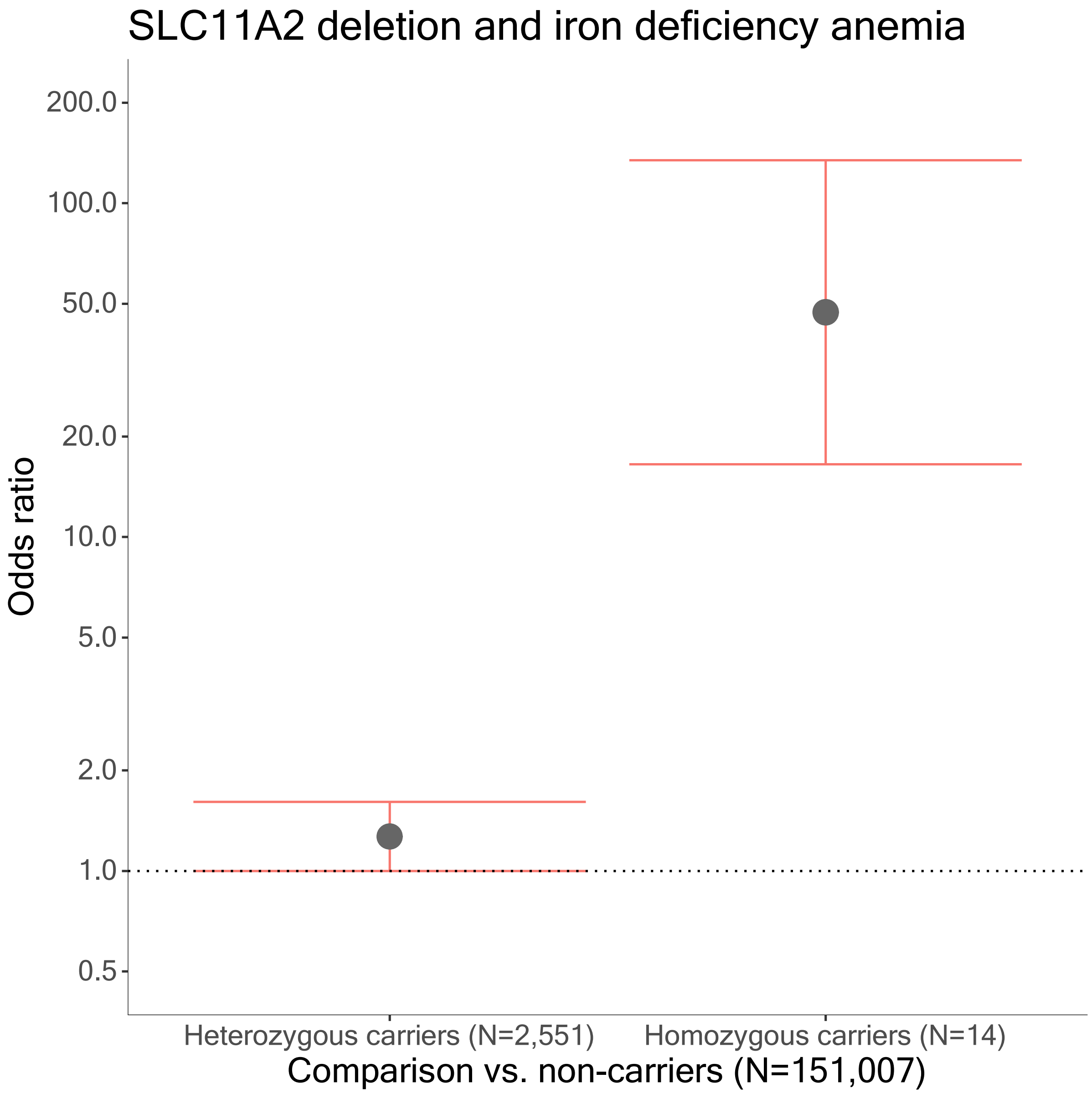

Supplementary Figure 7: The effects of SLC11A2 deletion genotype classes on iron deficiency anemia (IDA). The plot shows the odds ratio for IDA (error bars showing 95% confidence intervals) for heterozygous and homozygous SLC11A2 deletion carriers compared to wild-type.
