# Supplementary material for: A genome-wide meta-analysis yields 46 new loci associating with biomarkers of iron homeostasis
Source: Commun Biol. 2021 Feb 3;4:156. doi: 10.1038/s42003-020-01575-z (PMC7859200; doi:10.1038/s42003-020-01575-z)
Supplement: Supplementary file 3 — Description of Additional Supplementary Files [file 42003_2020_1575_MOESM3_ESM.pdf]

Description of Additional Supplementary Files

File Name: Supplementary Data

Description:

SUPPLEMENTARY DATA 1: Cohort summary statistics subdivided by sex and menopausal status for iron homeostasis parameters, hemoglobin, MCV and MCHC.

SUPPLEMENTARY DATA 2: Variants associated with ferritin, serum iron, iron binding capacity (TIBC) and/or transferrin saturation (TSAT) in the Icelandic/UK/Danish meta-analysis. Effects are measured in standard deviations (SD) and are always shown for the minor allele.

SUPPLEMENTARY DATA 3: Underlying data for variant-to-gene prediction algorithm.

SUPPLEMENTARY DATA 4: Genetic correlations between iron homeostasis biomarkers.

SUPPLEMENTARY DATA 5: Heritability (additive model) estimated for each phenotype (iron homeostasis biomarker) using parent-offspring regression and sibling regression.

SUPPLEMENTARY DATA 6: Sex-specific results for all genome-wide significant associations with ferritin, serum iron, iron binding capacity or transferrin saturation.

SUPPLEMENTARY DATA 7: Results for pre- vs. post-menopausal women for all genome-wide significant associations with ferritin, serum iron, iron binding capacity or transferrin saturation.

SUPPLEMENTARY DATA 8: Comparison of men and post-menopausal women for all genome-wide significant associations with ferritin, serum iron, iron binding capacity or transferrin saturation.

SUPPLEMENTARY DATA 9: Protein quantitative trait loci (pQTL) for iron homeostasis variants

SUPPLEMENTARY DATA 10: Sex-specific and pre vs postmenopausal associations for all coding variants in abnormal bleeding (HP:0001892) and venous thrombosis (HP:0004936) genes (listed in Human Phenotype Ontology (https://hpo.jax.org/)) with ferritin, serum iron, iron binding capacity or transferrin saturation.

SUPPLEMENTARY DATA 11: Effects on hemoglobin, mean corpuscular hemoglobin, mean corpuscular volume and reticulocytes counts in Iceland for the 62 variants that are associated with either ferritin, iron binding capacity, transferrin saturation or serum iron.

SUPPLEMENTARY DATA 12: Association of the 62 sequence variants found to associate with either ferritin, serum iron, iron binding capacity or transferrin saturation with the case-control phenotypes iron deficiency anemia and iron overload (transferrin saturation ever over 50%).

SUPPLEMENTARY DATA 13: Sex-specific results for iron deficiency anemia for the 62 variants associated with ferritin, serum iron, iron binding capacity or transferrin saturation. a and iron overload (transferrin saturation ever over 50%).

SUPPLEMENTARY DATA 14: Associations of the 62 iron homeostasis variants with the following clinical manifestations of iron deficiency or iron overload: restless legs, hemochromatosis, liver fibrosis/cirrhosis, liver cancer, type 2 diabetes, osteoarthritis, impotence, cardiomyopathy, osteoporosis, hyperpigmentation and amenorrhea.

SUPPLEMENTARY DATA 15: Association of ferritin and transferrin saturation (TSAT) polygenic risk scores (PRS) with the following clinical manifestations of iron deficiency or iron overload: restless legs, hemochromatosis, liver fibrosis/cirrhosis, liver cancer, type 2 diabetes, osteoarthritis, impotence, cardiomyopathy, osteoporosis, hyperpigmentation and amenorrhea.

SUPPLEMENTARY DATA 16: Differences in iron homeostasis markers and iron deficiency anemia in RLS cases vs controls.

SUPPLEMENTARY DATA 17: Clinical data for 13 homozygote carriers* of the SLC11A2 3.55kb deletion. Electronic medical records of carriers were reviewed for history of anemia and other major medical problems. (*one of 13 carriers does not live in Iceland, no medical records available)

SUPPLEMENTARY DATA 18: Sources for cis-eQTL data used in variant-to-gene algorithm.
