# Supplementary material for: A genome-wide meta-analysis yields 46 new loci associating with biomarkers of iron homeostasis
Source: Commun Biol. 2021 Feb 3;4:156. doi: 10.1038/s42003-020-01575-z (PMC7859200; doi:10.1038/s42003-020-01575-z)
Supplement: Supplementary file 5 — Reporting Summary [file 42003_2020_1575_MOESM5_ESM.pdf]

nature research

Corresponding author(s): Magnus K. Magnusson (magnus.magnusson@decode.is), Emanuele Di Angelantonio (ed303@medschl.cam.ac.uk), Henrik Ullum (Henrik.Ullum@regionh.dk), and Kari Stefansson (kstefans@decode.is)

Last updated by author(s): Sep 2, 2020

Reporting Summary

Nature Research wishes to improve the reproducibility of the work that we publish. This form provides structure for consistency and transparency in reporting. For further information on Nature Research policies, see our Editorial Policies and the Editorial Policy Checklist.

Statistics

For all statistical analyses, confirm that the following items are present in the figure legend, table legend, main text, or Methods section.

n/a	Confirmed	
☐	☒	The exact sample size (*n*) for each experimental group/condition, given as a discrete number and unit of measurement
☒	☐	A statement on whether measurements were taken from distinct samples or whether the same sample was measured repeatedly
☐	☒	The statistical test(s) used AND whether they are one- or two-sided<br>*Only common tests should be described solely by name; describe more complex techniques in the Methods section.*
☐	☒	A description of all covariates tested
☐	☒	A description of any assumptions or corrections, such as tests of normality and adjustment for multiple comparisons
☐	☒	A full description of the statistical parameters including central tendency (e.g. means) or other basic estimates (e.g. regression coefficient) AND variation (e.g. standard deviation) or associated estimates of uncertainty (e.g. confidence intervals)
☐	☒	For null hypothesis testing, the test statistic (e.g. *F*, *t*, *r*) with confidence intervals, effect sizes, degrees of freedom and *P* value noted<br>*Give P values as exact values whenever suitable.*
☒	☐	For Bayesian analysis, information on the choice of priors and Markov chain Monte Carlo settings
☒	☐	For hierarchical and complex designs, identification of the appropriate level for tests and full reporting of outcomes
☐	☒	Estimates of effect sizes (e.g. Cohen's *d*, Pearson's *r*), indicating how they were calculated

Our web collection on statistics for biologists contains articles on many of the points above.

Software and code

Policy information about availability of computer code

Data collection

We used publicly available software (URLs listed below) in conjunction with the above described algorithms in the sequencing processing pipeline (Whole-genome sequencing, Association testing, RNA-seq mapping and analysis):
BWA 0.7.10 mem, https://github.com/lh3/bwa
GenomeAnalysisTKLite 2.3.9, https://github.com/broadgsa/gatk/
Picard tools 1.117, https://broadinstitute.github.io/picard/
SAMtools 1.3, http://samtools.github.io/
Bedtools v2.25.0-76-g5e7c696z, https://github.com/arq5x/bedtools2/
Variant Effect Predictor https://github.com/Ensembl/ensembl-vep
BOLT-LMM https://data.broadinstitute.org/alkesgroup/BOLT-LMM/downloads/"
IMPUTE2 v2.3.1 https://mathgen.stats.ox.ac.uk/impute/impute_v2.html
dbSNP v140; http://www.ncbi.nlm.nih.gov/SNP/
BiNGO v3.0.3 https://www.psb.ugent.be/cbd/papers/BiNGO/Download.html
Cytoscape v3.7.1 https://cytoscape.org/download.html

We used R extensively to analyze data and create plots.

Data analysis

We used publicly available software (URLs listed below) in conjunction with the above described algorithms in the sequencing processing pipeline (Whole-genome sequencing, Association testing, RNA-seq mapping and analysis):
BWA 0.7.10 mem, https://github.com/lh3/bwa
GenomeAnalysisTKLite 2.3.9, https://github.com/broadgsa/gatk/
Picard tools 1.117, https://broadinstitute.github.io/picard/

SAMtools 1.3, http://samtools.github.io/
Bedtools v2.25.0-76-g5e7c696z, https://github.com/arq5x/bedtools2/
Variant Effect Predictor https://github.com/Ensembl/ensembl-vep
BOLT-LMM https://data.broadinstitute.org/alkesgroup/BOLT-LMM/downloads/"
IMPUTE2 v2.3.1 https://mathgen.stats.ox.ac.uk/impute/impute_v2.html
dbSNP v140; http://www.ncbi.nlm.nih.gov/SNP/
BiNGO v3.0.3 https://www.psb.ugent.be/cbd/papers/BiNGO/Download.html
Cytoscape v3.7.1 https://cytoscape.org/download.html

We used R extensively to analyze data and create plots.

For manuscripts utilizing custom algorithms or software that are central to the research but not yet described in published literature, software must be made available to editors and reviewers. We strongly encourage code deposition in a community repository (e.g. GitHub). See the Nature Research guidelines for submitting code & software for further information.

Data

Policy information about availability of data

All manuscripts must include a data availability statement. This statement should provide the following information, where applicable:

- Accession codes, unique identifiers, or web links for publicly available datasets
- A list of figures that have associated raw data
- A description of any restrictions on data availability

The Icelandic population WGS data has been deposited at the European Variant Archive under accession code PRJEB15197. The authors declare that the data supporting the findings of this study are available within the article, its Supplementary Data files and upon request. Overall meta-analysis summary statistics have been shared at https://www.decode.com/summarydata/. The UK Biobank data can be obtained upon application (ukbiobank.ac.uk)

Field-specific reporting

Please select the one below that is the best fit for your research. If you are not sure, read the appropriate sections before making your selection.

[x] Life sciences [ ] Behavioural & social sciences [ ] Ecological, evolutionary & environmental sciences

For a reference copy of the document with all sections, see nature.com/documents/nr-reporting-summary-flat.pdf

Life sciences study design

All studies must disclose on these points even when the disclosure is negative.

Sample size	The sample sizes for each cohort correspond to all available data from Denmark (Danish Blood Donor Study), Iceland and UK (Interval Study) where genotype data and iron homeostasis biomarkers levels were available, after quality control as specified in the Methods section.
Data exclusions	No data exclusion
Replication	Three independent cohorts from Iceland, UK and Denmark were used in this meta-GWAS. Test for heterogeneity between populations of the effect for all variants was carried out. When rare variants were only present in one population (and no replication was available) this was discussed specifically.
Randomization	No randomization was used.
Blinding	Not relevant for this study, as this is a GWAS meta-analysis study.

Reporting for specific materials, systems and methods

We require information from authors about some types of materials, experimental systems and methods used in many studies. Here, indicate whether each material, system or method listed is relevant to your study. If you are not sure if a list item applies to your research, read the appropriate section before selecting a response.

Materials & experimental systems

n/a	Involved in the study
☒	☐ Antibodies
☒	☐ Eukaryotic cell lines
☒	☐ Palaeontology and archaeology
☒	☐ Animals and other organisms
☐	☒ Human research participants
☒	☐ Clinical data
☒	☐ Dual use research of concern

Methods

n/a	Involved in the study
☒	☐ ChIP-seq
☒	☐ Flow cytometry
☒	☐ MRI-based neuroimaging

Human research participants

Policy information about studies involving human research participants

Population characteristics	Study subjects from Iceland<br>The Icelandic data (where around one half of all individuals had repeated measurements) include over half the adult population in Iceland and the vast majority of all clinical laboratory results in Iceland from 1990-2017. Participants were recruited with informed consent as part of various genetic population studies at deCODE genetics, Reykjavík, Iceland.<br>Study subjects from UK<br>The INTERVAL study is a prospective cohort study of approximately 45,000 blood donors, representative of the wider donor population, nested in a randomized control trial. Participants, aged 18 years or older, were recruited between 2012 and 2014 from 25 National Health Service Blood and Transplant static donor centers in England.<br>Study subjects from Denmark<br>The Danish Blood Donor Study (DBDS), initiated in 2010 as a collaborative blood donor oriented and generic research platform and is an on-going nation-wide prospective cohort with inclusion sites at all Danish blood collection facilities. Currently, more than 110,000 blood donors are participating, and more than 95% of invited blood donors are willing to participate [75]. Due to the step-wise roll-out of DBDS, an enrichment of individuals from the greater Copenhagen region (the capital) and the central region of Jutland (the second largest city) are present in this study.
Recruitment	See above
Ethics oversight	Iceland: All participants who donated samples gave informed consent and the National Bioethics Committee of Iceland approved the study (VSN-15-198) which was conducted in agreement with conditions issued by the Data Protection Authority of Iceland. Personal identities of the participant's data and biological samples were encrypted by a third-party system (Identity Protection System), approved and monitored by the Data Protection Authority.<br>UK: . All participants in UK Interval study provided written, informed consent and the study was approved by the Cambridge (East) Research Ethics Committee (ref: 11/EE/0538).<br>Denmark: The Danish Blood Donor Study has secured necessary permissions and approval from the Danish Data Protection Agency (2007-58-0015) and the Scientific Ethical Committee system (M-20090237).<br>Use of data from UK Biobank was through an application by deCODE Genetics under UKB project number 56270.

Note that full information on the approval of the study protocol must also be provided in the manuscript.
